# Luteibacter mycovicinus sp. nov., a yellow-pigmented gammaproteobacterium found as an endohyphal symbiont of endophytic Ascomycota

**DOI:** 10.1099/ijsem.0.006240

**Published:** 2024-05-02

**Authors:** David A. Baltrus, Morgan Carter, Meara Clark, Caitlin Smith, Joseph Spraker, Patrik Inderbitzin, A. Elizabeth Arnold

**Affiliations:** 1 School of Plant Sciences, The University of Arizona, Tucson, AZ 85721, USA; 2 School of Animal and Comparative Biomedical Sciences, The University of Arizona, Tucson, AZ 85721, USA; 3 Indigo Agriculture, Boston, Massachusetts, USA; 4 Department of Ecology and Evolutionary Biology, The University of Arizona, Tucson, AZ 85721, USA

**Keywords:** endohyphal bacteria, EHB, endophyte, *Luteibacter mycovicinus*

## Abstract

We isolated and described a yellow-pigmented strain of bacteria (strain 9143^T^), originally characterized as an endohyphal inhabitant of an endophytic fungus in the Ascomycota. Although the full-length sequence of its 16S rRNA gene displays 99 % similarity to *Luteibacter pinisoli*, genomic hybridization demonstrated <30 % genomic similarity between 9143^T^ and its closest named relatives, further supported by average nucleotide identity results. This and related endohyphal strains form a well-supported clade separate from *L. pinisoli* and other validly named species including the most closely related *Luteibacter rhizovicinus*. The name *Luteibacter mycovicinus* sp. nov. is proposed, with type strain 9143^T^ (isolate DBL433), for which a genome has been sequenced and is publicly available from the American Type Culture Collection (ATCC TSD-257^T^) and from the Leibniz Institute DSMZ (DSM 112764^T^). The type strain reliably forms yellow colonies across diverse media and growth conditions (lysogeny broth agar, King’s Medium B, potato dextrose agar, trypticase soy agar and Reasoner's 2A (R2A) agar). It forms colonies readily at 27 °C on agar with a pH of 6–8, and on salt (NaCl) concentrations up to 2 %. It lacks the ability to utilize sulphate as a sulphur source and thus only forms colonies on minimal media if supplemented with alternative sulphur sources. It is catalase-positive and oxidase-negative. Although it exhibits a single polar flagellum, motility was only clearly visible on R2A agar. Its host range and close relatives, which share the endohyphal lifestyle, are discussed.

## Data Summary

Supplementary files available in the online version of this article can be found at Figshare 10.6084/m9.figshare.16528950.v1 [1].

## Introduction

Fungal phenotypes, including tolerance to stress and plant growth promotion capabilities, can be altered by bacteria that occur within living fungal hyphae (endohyphal bacteria; EHB) [2]. Although many well characterized EHB are transmitted vertically between fungal generations and include many with obligately symbiotic lifestyles [3,5], recent studies reveal that fungi can be inhabited by diverse, facultative, and horizontally transmitted EHB [2]. These symbionts generally have relatively large genomes (3.9–8.5 Mb) and have diverse mechanisms for interacting with fungal cells [2,6]. Many can be isolated in culture and may emerge from living cultures of fungi under common laboratory conditions [7]. One clade of *Luteibacter* strains has been repeatedly found within the hyphae of endophytic Ascomycota [7,8]. The genus *Luteibacter* has few named species and contains typically yellow-pigmented bacteria predominately associated with plants and soils. The first member of the genus, *Luteibacter rhizovicinus*, and *Luteibacter pinisoli* were isolated from the rhizospheres of barley [9] and pine trees [10], respectively. Similarly, *Luteibacter yeojuensis* was isolated from greenhouse soil [11,12]. However, additional species have been found in human blood (*Luteibacter anthropi* [12]) and industrial factories (*Luteibacter jiangsuensis* [13]), and studied for unique metabolic attributes [14]. Beyond these named species, there are >10 unnamed species of *Luteibacter* represented in the Genome Taxonomy DataBase (GTDB). Here we characterize a type specimen, strain 9143^T^, and propose the species name *Luteibacter mycovicinus* for the unnamed clade of endohyphal *Luteibacter* strains.

## Isolation and ecology

Bacterial strain 9143^T^was isolated from living hyphae of a foliar fungal endophyte (*Pestalotiopsis* sp. 9143^T^, Pezizomycotina, Ascomycota, with affinity for *Pe. neglecta*), which itself was isolated on a standard growth medium (2 % malt extract agar) from surface-sterilized, apparently healthy foliage of the conifer *Platycladus orientalis* (Cupressaceae) [7]. Bacterial strain 9143^T^emerged initially from its fungal host after heat shock at 36 °C and grew as a yellow-pigmented colony on potato dextrose agar (PDA) [15]. For characterization, single colonies of bacterial strain 9143^T^ were isolated on lysogeny broth agar (LBA), with colonies appearing after 3 days of growth at 27 °C. An individual colony from this isolation (DBL433) was grown overnight in lysogeny broth (LB) and frozen in the Baltrus Lab collection. This strain was characterized initially as *Xanthomonadaceae* sp. (*Gammaproteobacteria*) [7] and subsequently as *Luteibacter* sp. based on phylogenetic analyses of the 16S rRNA gene [16]. Strain 9143^T^ is a member of a clade of endohyphal *Luteibacter* strains encountered in diverse fungal endophytes, including strains 9133 and 9145, that were similarly isolated as part of the same set of studies [6,7, 16]. Previous studies demonstrated that fungal hosts can be cleared of bacterial strains by antibiotic treatment [15,16]. Strain 9143^T^ also can be introduced into diverse endophytic fungi, including the original fungal host as well as members of other classes of filamentous *Ascomycota* [15].

## Genome features

**Fig. 1.**
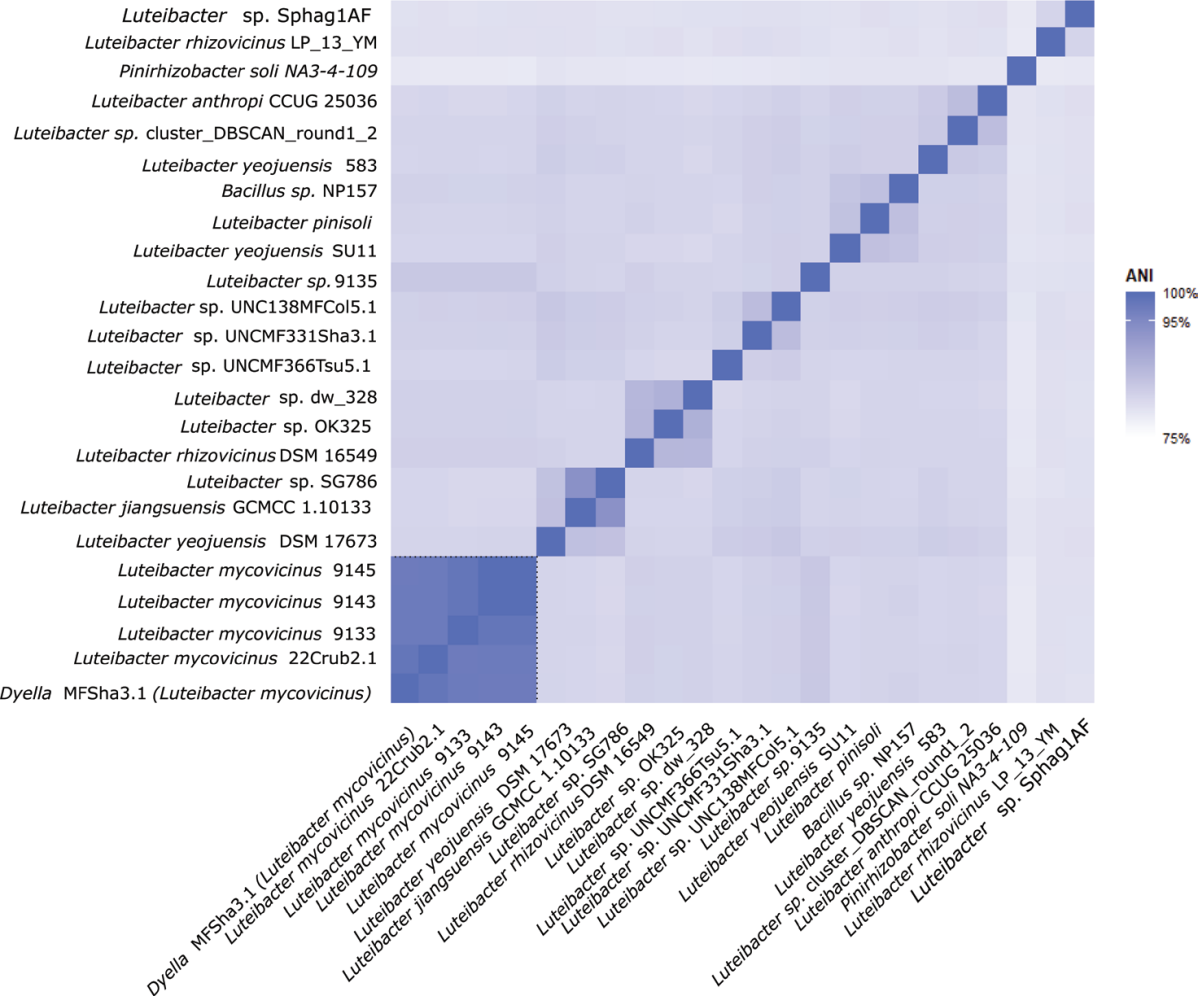
ANI values for *Luteibacter mycovicinus* strains compared to related strains at the Genome Taxonomy DataBase (GTDB). All *Luteibacter* species representatives were included and are labelled with their NCBI taxonomy name. A dotted line indicates the five strains within one species-level group (>95 % ANI) that includes *L. mycovicinus* 9143^T^.

The complete genome sequence of type strain 9143^T^ was reported previously as part of a comparative study with other EHB [6], including strains 9133 and 9145, and is available at GenBank (accession GCA_000745235.1). The genome sequence of strain 9143^T^ is 4 625 266 bp in length [6]. It contains a G+C content of 64.84 %, 4143 annotated protein-coding genes, six annotated rRNA genes, and 50 annotated tRNA genes [6]. We used the GTDB FastANI interface to load in all species representatives for *Luteibacter* and then identified all other strains with >95 % identity to the proposed type strain (Fig. 1). Within the GTDB, there are four additional strains with greater than 95 % identity with our type strain as calculated by average nucleotide identity (ANI) analysis, two of which are 9145 and 9133 that were isolated and sequenced within the same studies [6,7]. None of these strains has been previously given a formal species name. They fall under the species representative *Luteibacter* sp. 9145 at GTDB, which has a genome that is nearly identical to 9143^T^ but is missing a putative prophage. The other strains are named *Dyella* sp. 333MFSha (which we propose to reclassify as *Luteibacter*) and *Luteibacter* sp. 22Crub2.1, both of which were isolated in studies of plant root communities (JGI GOLD Project IDs Gp0115192 and Gp0024616).

Of the five strains proposed to be *L. mycovicinus* sp. nov., all have genomes between 4.5–4.7 Mbp and with similar gene content as strain 9143^T^. Prediction of the putative secondary metabolite clusters by the programme antiSMASH 7.0 using default parameters [17] showed conserved pathways for uncharacterized lanthipeptides and arylpolyenes, as well as a novel interleaved polyketide synthase and nonribosomal peptide synthase cluster (Table 1). Strains 9133, 9143^T^, 9145 and 22Crub2.1 were all previously found to have Type II, IV, VI and I secretion systems as well as the Sec and Tat pathways [6]; 22Crub2.1 likely has an additional Type IV and VI secretions systems, and 9133 an additional Type IV, compared to 9143^T^.

**Table 1.** Secondary metabolite clusters present in the genomes of proposed *Luteibacter mycovicinus* sp. nov. strains

Putative cluster type:	**9143^T^**	**9145**	**9133**	22Crub2.1	333MFSha
Lanthipeptide Class I	−	−		+	−
Lanthipeptide Class II	+	+	+	+	+
Lanthipeptide Class III	+	+	−	+	+
Lanthipeptide Class IV	+	+	++	+	
Type I PKS/NRPS	+	+		+	+
Arylpolyene (Xanthomonadin I-like)	+	+	+	+	+
Arylpolyene	+	+	+	+	+
RiPP-like	+	+	+	+	+

## ANI and whole genome phylogeny

Strain 9143^T^ shares <83 % ANI with representatives strains for other named *Luteibacter* species (Fig. 1), including *L. rhizovicinus* and *L. pinsoli*, falling short of the typical species threshold of 95 % [8]. We also note that, according to ANI classification, the next closest validly named species to *Luteibacter* sp. 9143^T^ is *L. rhizovicinus* DSM 16549^T^, but also that there are multiple strains classified as *L. rhizovicinus* at GTDB that can themselves be separated by ANI values into two genomic species.

**Fig. 2.**
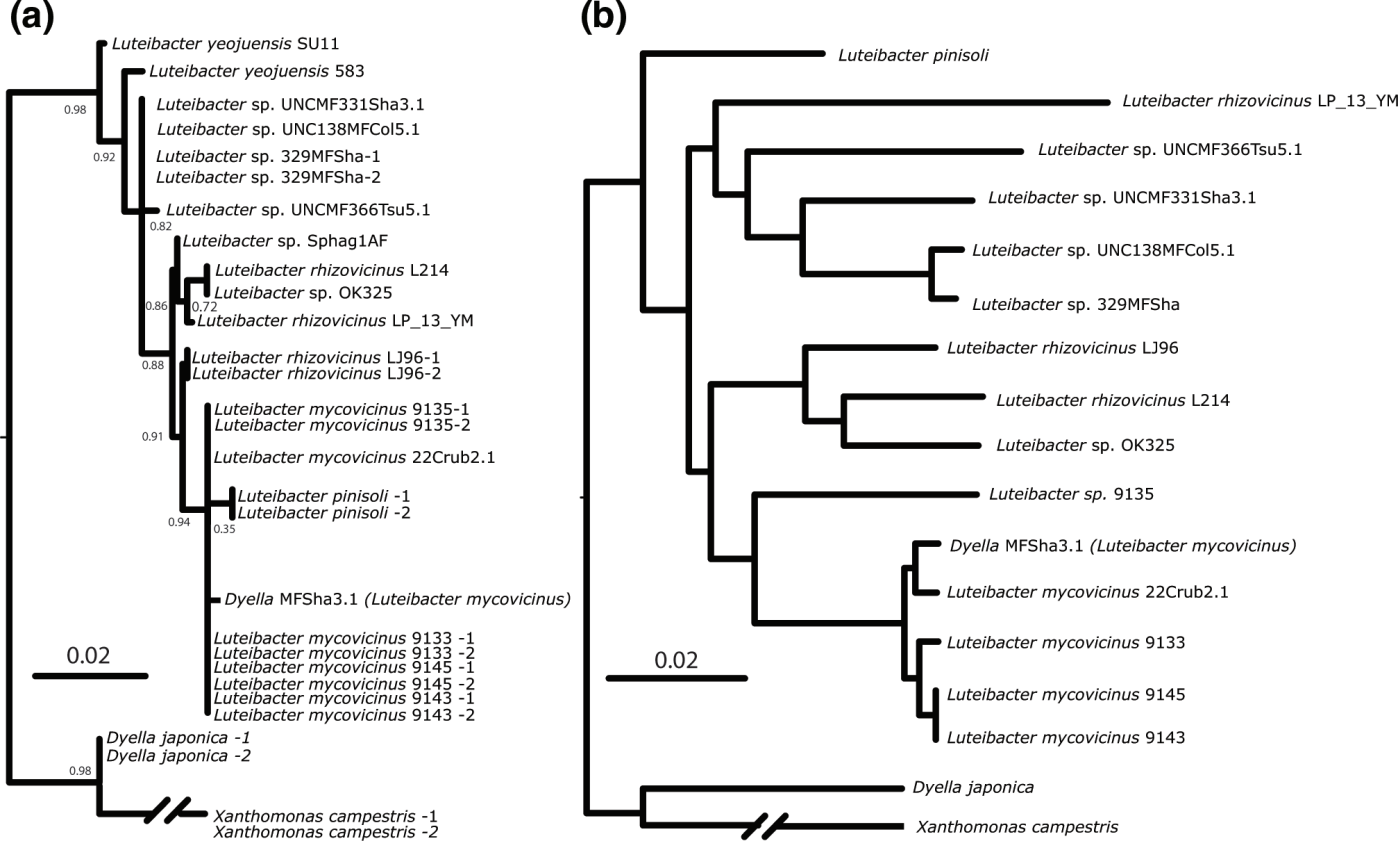
Phylogenetic placement of *Luteibacter mycovicinus* sp. nov. within the genus *Luteibacter*. Phylogenies were inferred using (a) nearly full-length copies of 16S rRNA using RaxML-ng. Bootstrap support values are shown for informative nodes, with the remaining unlabeled nodes either being >90 % bootstrap values (for multiple copies within a single genome) or <75 % for all other nodes. Where possible, additional 16S rRNA gene copies were included for each genome and are denoted by a dash (–) followed by either ‘1’ or ‘2’. (**b**) Whole genome polymorphisms using RealPhy which is based on the maximum-likelihood method.

Phylogenomic analysis using the RealPhy server [18], where *Xanthomonas campestris*, *Luteibacter* sp. 9143^T^, and *Luteibacter pinisoli* were used as references, confirmed placement in *Xanthomonadaceae* (*Xanthomonadales*, *Gammaproteobacteria*) [6]. In contrast to the results from 16S rRNA gene classification, taxonomic assessment of *Luteibacter* strains using both the ANI values (Fig. 1) and based on polymorphisms throughout the genome (Fig. 2b) strongly suggests that *L. pinisoli* forms a separate taxonomic clade from strains we propose to form *L. mycovicinus* sp. nov. and typified by strain 9143^T^ (Figs1  2b).

To confirm that *L. mycovicinus* sp. nov. was a separate species from the most closely related, validly named species based on genomic and phylogenetic data*,* we carried out DNA–DNA hybridizations with a type strain of *L. rhizovicinus* (LJ96, ATCC BAA-1015, hereafter, DBL1164). These analyses confirmed our genome sequence level evaluations and demonstrated that strain 9143^T^ can be considered a different species (<30 % relatedness). DNA–DNA hybridizations were carried out by DSMZ, and the report, as well as methods for this characterization, can be found on Figshare [19].

## 16S rRNA is not a precise classifier for *Luteibacter* species

Despite the evidence from ANI and whole genome phylogeny that *Luteibacter* strain 9143^T^ and related strains represent a new species, the 16S rRNA sequence is >99 % similar to named species *L. pinisoli*. To address the value of general classification of *Luteibater* species by 16S rRNA gene sequences, sequences for full length 16S rRNA loci were obtained from JGI IMG for a larger set of *Luteibacter* species and related strains, with all copies of this locus included where possible. We have deposited full length 16S rRNA sequences for *Luteibacter* strains 9133, 9143^T^ and 9145 at NCBI under accession numbers ON786549.1, ON786547.1 and ON786548.1, respectively.

For phylogenetic inference based on 16S rRNA genes, sequences were aligned using Clustal Omega with default parameters [20]. A file containing aligned sequences can be found at Figshare [21]. Ends of the sequence alignments were trimmed so that alignments from all sequences started and stopped at the same position. The evolutionary model with the best fit to the data (TPMuf +I) was chosen using modeltest-ng 0.1.6 [22]. The topology was inferred via RaxML-ng [23]. Bootstrap analyses displayed convergence after 3500 trees (see convergence file [21]), at which point bootstrap values were summed using the -tbe function of RaxML-ng.

Overall, across the genus *Luteibacter*, there is little divergence in the 16S rRNA gene (with aligned sequence comparisons displaying >98 % identity) (Fig. 2a, File S1, available in the online version of this article and [21]). Moreover, phylogenies inferred from entire 16S rRNA gene sequences for *Luteibacter* species place *L. pinisoli* as a nested branch inside a clade containing all other strains proposed to be *L. mycovicinus*, although confidence intervals across the tree are relatively low due to the lack of informative sites (Fig. 2a). It is important to note that relationships between these strains inferred from the 16S rRNA gene alone are consistent regardless of which genomic copy is used, as sequences found within the same genome are usually identical (Fig. 2a). However, as demonstrated with ANI analysis and whole genome-based phylogeny, 16S rRNA gene sequence comparisons do not provide an accurate representation of potential species groupings in *Luteibacter* and thus we do not believe that *L. pinisoli* and *L. mycovicinus* sp. nov. are as closely related as the 16S rRNA gene phylogeny suggests.

## Physiology and chemotaxonomy

Strain 9143^T^ was analysed for growth on a variety of nutrient sources by Biolog using their standard workflows and pipelines for phenotype microarray plates PM1, PM3B and PM4A [24]. Results of these assays suggest that this strain can utilize numerous carbon, nitrogen and phosphorus sources [25]. Along this metabolic characterization, we have also previously demonstrated an inability for strain 9143^T^ to grow in minimal media where sulphate is the sole sulphur source [26]. However, organic sulphur sources (cysteine and methionine), some inorganic sources (thiosulphate), and the presence of the fungal host enable growth in minimal medium [26,27]. It can form colonies on M9 minimal media agar supplemented with casamino acids (0.1 % w/v) after 3–5 days at 27 °C (Fig. 1).

**Fig. 3.**
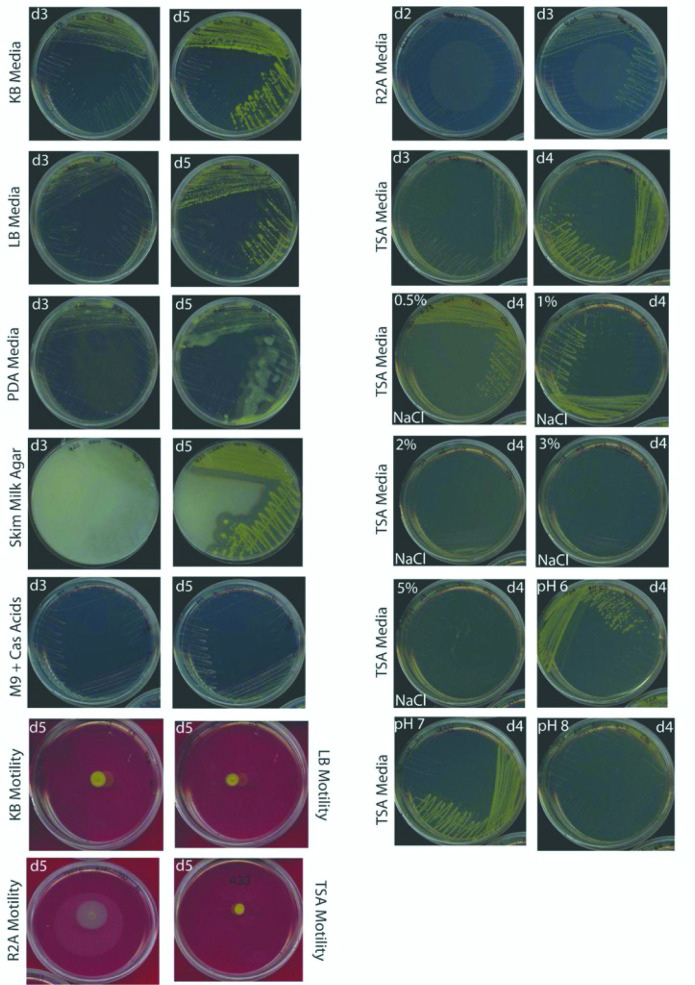
Representative photos of *Luteibacter mycovicinus* strain 9143^T^ on various media. Days of growth are indicated in the corner of each photo with d followed by a number. Plates were incubated at 27 °C.

Strain 9143^T^ forms colonies on LBA, King’s Medium B (KB) agar, Reasoner’s 2A (R2A) agar, PDA and trypticase soy agar (TSA) after 3–5 days at 27 °C (Fig. 3). Colonies on KB agar appear more mucoid than on other media. On PDA it displays a mucoid and spreading morphology. The strain displays clearing zones after 3–5 days of growth on skimmed milk agar (Fig. 3).

We examined motility of strain 9143^T^ on motility agar (0.5× of each medium with 0.25 % agar). Its genome contains genes and pathways implicated in flagellum formation [8], but it was motile only when stabbed into R2A motility agar. No motility was observed when it was stabbed into KB, LB, or TSA motility agar (Fig. 3).

We tested for growth of strain 9143^T^ on TSA at a wide range of pH values, adjusted with either HCl or NaOH. The strain can form colonies at pH 6–8 but not pH 9 (Fig. 3). Likewise, we tested for the ability of strain 9143^T^ to form colonies in TSA plates containing different levels of salt (NaCl). This strain displays growth at 0.5, 1 and 2 % (w/v) salt but not 3 or 5 % (Fig. 3).

Strain 9143^T^ was positive for catalase activity as bubbles rapidly formed when a 3 % solution of hydrogen peroxide was added to colonies grown on KB media [28]. We considered strain 9143^T^ negative for oxidase activity because it took >15 s for a blue colour to appear when cells were streaked to standard oxidase test discs (Sigma Aldrich) containing *N*,*N*-dimethyl-*p*-phenylenediamine oxalate and α-naphthol.

Respiratory quinone, fatty acid composition and polar lipid characterizations were carried out by Identification Service and Dr. Brian Tindall (DSMZ, Braunschweig, Germany) [19]. Cells were grown overnight in LB media at 27 °C and freeze-dried before shipment. Ubiquinone 8 (Q8) was identified as a respiratory quinone, and the fatty acid composition of strain 9143^T^ consisted mainly of C_15 : 0_ iso (21.60 %), C_16 : 0_ (10.75 %), C_17.1_ isoω8*c* (10.71 %) and C_17 : 0_ iso (19.03 %). Aminolipid, phospholipid, phosphatidylglycerol and phosphatidylethanolamine were identified as polar lipids.

## Microscopy

**Fig. 4.**
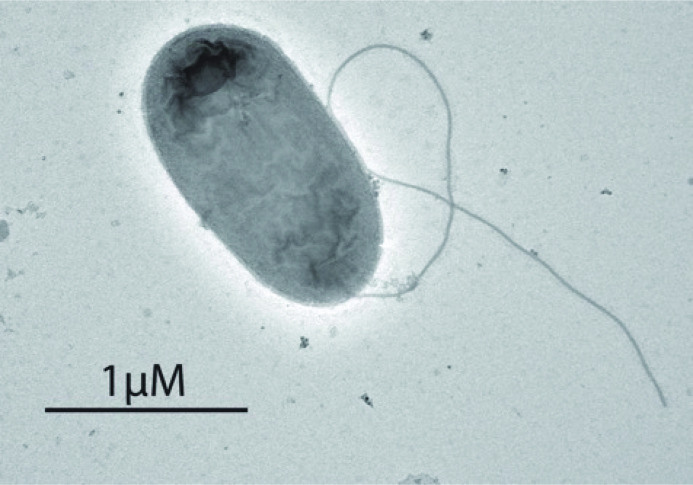
Transmission electron microscopy image of *Luteibacter mycovicinus* strain 9143^T^.

Strain 9143^T^ was grown overnight in R2A media at 27 °C, pelleted by spinning for 4 min at 2000 r.p.m., and resuspended in distilled water for transmission electron microscopy. Formvar-coated 150 mesh copper grids were floated on a drop of suspension for 2 min. Excess suspension was aspirated using filter paper held against the edge of each grid, leaving a thin film. Grids then were floated on 2 % aqueous uranyl acetate for 1 min and the excess aspirated as before. Grids were viewed in an FEI T12 BioTwin electron microscope operated at 100kv. Eight-bit Tiff images were collected via an AMT XR41 CCD camera. In Fig. 4, a representative microscopy image of strain 9143^T^ depicts a rod-shaped bacterium with a single polar flagellum.

**Fig. 5.**
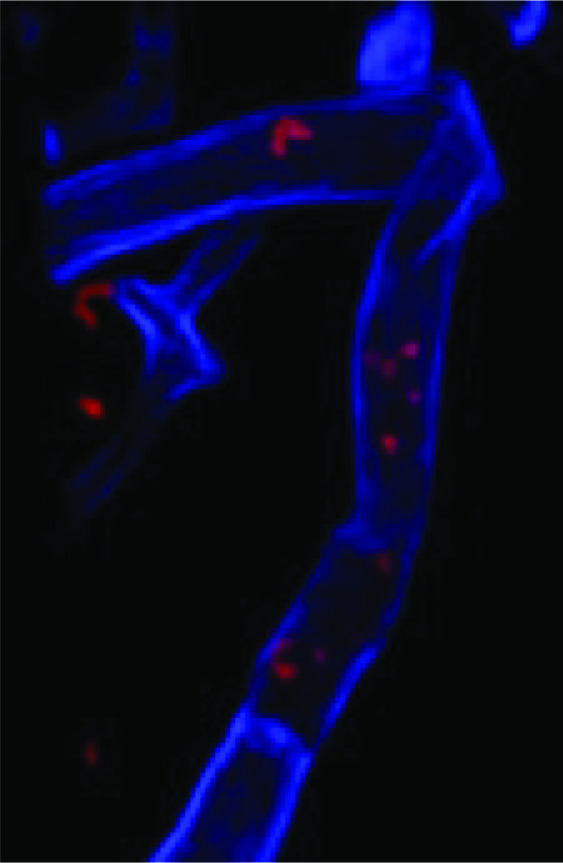
3D projection of a Calcofluor White-stained fungal hyphae of *Pestalotiopsis* sp. 9143^T^ in blue with *Luteibacter mycovicinus* expressing tdTomato in red. Full video can be found as File S1.

A sample of strain 9143^T^ with integrated tdTomato constitutively driven by P*_lac_* (DBL920) [15] was reassociated with *Pestalotiopsis* sp. 9143^T^ as described previously [29]. After culturing in minimal media, fungal tissue was stained by submerging in Calcofluor White at a concentration of 1 mg ml^−1^ for 5 min prior to mounting. Tissue was imaged within an hour on a Zeiss LSM880 inverted confocal microscope using the Immersion Oil Plan-Apochromat ×63/1.40 oil M27 lens. Z-stacks were taken with a 0.5 µm distance between stacks using 561/631 nm and 405/467 nm channels to capture the tdTomato and Calcofluor White signal, respectively. The 3D projection shown in Fig. 5 was processed using Fiji and only a single plane was used for both channels.

## Description of *Luteibacter mycovicinus* sp. nov.

*Luteibacter mycovicinus* (my.co.vi.ci’nus. Gr. masc. n. *mykes*, fungus; L. masc. adj. *vicinus*, neighbouring; N.L. masc. adj. *mycovicinus*, neighbouring a fungus, referring to the original isolation of this strain as a facultative endohyphal associate of a fungus).

Displays the following properties. Yellow colonies, 3–5 mm in diameter and with an entire edge after 48 h at 27 °C when grown on LBA. The main fatty acids are C_15 : 0_ iso, C_16 : 0_, C_17.1_ isoω8*c* and C_17 : 0_ iso. The type strain is 9143^T^ (DBL433), isolated after emergence from a foliar fungal endophyte identified as *Pestalotiopsis* sp. 9143^T^, which was isolated from the foliage of the conifer *Platycladus orientalis* (Cupressaceae) in Durham, NC, USA. The complete genome sequence of this strain was reported previously [6] and can be found at GenBank (accession: GCA_000745235.1); a separate 16S rRNA sequence entry is available as well (accession: ON786547). The strain has been deposited at the American Type Culture Collection (ATCC TSD-257^T^) and the DSMZ (DSM 112764^T^).

## Abbreviations

ANI: average nucleotide identity
EHB: endohyphal bacteria
GTDB: Genome Taxonomy DataBase
LBA: lysogeny broth agar
PDA: potato dextrose agar
